# Distancing Adherence and Negative Emotions among the Israeli Elderly Population during the COVID-19 Pandemic

**DOI:** 10.3390/ijerph18168770

**Published:** 2021-08-19

**Authors:** Shiran Bord, Ayelet Schor, Carmit Satran, Ola Ali Saleh, Liron Inchi, Dafna Halperin

**Affiliations:** 1 Health Systems Management Department, The Max Stern Yezreel Valley College, Yezreel Valley 1930600, Israel; ayelets@yvc.ac.il (A.S.); Olaa@yvc.ac.il (O.A.S.); Lironi@yvc.ac.il (L.I.); Dafnah@yvc.ac.il (D.H.); 2 Nursing Department, The Max Stern Yezreel Valley College, Yezreel Valley 1930600, Israel; carmits@yvc.ac.il

**Keywords:** COVID-19, distancing adherence, older people, trust in healthcare system, social support, negative emotion

## Abstract

Social distancing was found to prevent COVID-19 contagion. Therefore, understanding the factors associated with the public’s adherence is important. Acknowledging the importance of emotional wellbeing regarding older people’s health, and understanding their emotional state during the pandemic, are crucial. This study assessed factors associated with older people’s adherence to social distancing and their emotional status. A cross-sectional online survey was conducted among 1822 respondents above the age of 60. Distancing adherence, negative emotion, trust, social support, threat perception, attitudes, and subjective norms were assessed, and a path analysis was performed. Adherence was positively associated with attitudes (β = 0.10; *p* < 0.001), and with subjective norms (β = 0.19; *p* < 0.001). Negative emotions were positively associated with threat perception (β = 0.33; *p* < 0.001), and negatively associated with social support (β = −0.13; *p* < 0.001) and subjective norms (β = −0.10; *p* < 0.001). Attitudes mediated the relationship of threat perception (95% CI = 0.009, 0.034), trust (95% CI = 0.008, 0.029), and social support (95% CI = 0.006, 0.023) with distancing adherence. Subjective norms mediated the relationship between threat perception (95% CI = 0.014, 0.034), trust (95% CI = 0.026, 0.055), and social support (95% CI = 0.002, 0.048) with distancing adherence. Subjective norms mediated the relationship between threat perception (95% CI = −0.022, −0.006), trust (95% CI = −0.034, −0.010), and social support (95% CI = −0.029, −0.009) with negative emotions. When promoting social distancing adherence, subjective norms and attitudes must be considered, as they play a role in promoting adherence and negative-emotion regulation.

## 1. Introduction

COVID-19 is an infectious disease transmitted through small droplets and human touch. It was declared a global pandemic by the WHO in March 2020 [1]. According to data currently accumulated globally, people over 60 years of age are among the populations with the highest risk of COVID-19 morbidity, complications, and mortality [2].

In order to prevent the public from contracting the disease and contain the epidemic, a number of guidelines were issued by the WHO, including social distancing and isolation [1]. A qualitative study conducted in the United Kingdom pointed out that although participants tended to follow the instructions, they also reported knowing many people who did not adhere to them. Moreover, the participants described social distancing and isolation as having a substantial negative impact on their wellbeing [3].

The importance of adhering to health recommendations was demonstrated in Reuben et al.’s (2020) study, which indicated that 19.7% of the change in COVID-19 community transmission was accounted for by community adherence to governmental recommendations [4].

Adhering to health recommendations is a conscious behavioral choice that is propelled, among other things, by one’s trust in the healthcare system. Studies have demonstrated that reduced trust in this system was associated with lower adherence [5,6].

Furthermore, peoples’ perception of the required behavior and its consequences is crucial. The Health Belief Model (HBM) [7] discusses people’s perceived susceptibility to the disease and its perceived severity, describing to what extent a person believes they are susceptible to the disease or its consequences, and the levels of severity a person associates with the disease and/or its consequences. The model also points to the perceived benefit a person finds in the required health behavior. According to the HBM, high perceptions of susceptibility, severity, and benefit promote the required behavior. Ajzen and Fishbein’s Theory of Reasoned Action (TRA) [8] expands the HBM, adding attitudes, intentions, and perceived social norms as fundamental in predicting goal-directed behaviors [9]. According to the TRA, people’s attitudes and the way they perceive the social norms related to the required behavior may predict their behavioral intentions, as well as their behavior itself.

A recent systematic review examining factors associated with older people’s adherence to seasonal influenza vaccinations pointed to threat perception, behavioral beliefs, and subjective norms as key factors in predicting adherence [10]. Furthermore, a study conducted in Denmark that examined willingness to distance during the COVID-19 pandemic identified threat perception (how threatening to the nation one perceives the pandemic to be) as a significant predictor of one’s willingness to distance [11].

Adhering to recommendations during the COVID-19 pandemic requires keeping one’s distance from family and friends for a long period of time. Distancing may impair a person’s wellbeing and increase negative emotions, such as stress and anxiety, especially at a time of substantial uncertainty and the inability to predict the future [12]. This is significant, as a negative emotional state is known to have a negative effect on people’s mental and physical health, especially among older people, as the association between negative emotions and chronic physical illness was found to increase with age [13]. Moreover, according to the Emotion Imbued Choice (EIC) model [14], negative emotions, such as fear and anxiety regarding outcomes, may evoke a greater appraisal of risk and uncertainty, which may influence judgment and decision-making processes [15].

While the HBM and TRA do not include emotions as an underlying construct, it is assumed that emotions’ ability to influence behavior stems from their ability to gather energy in order to act or avoid action [16]. Therefore, UI-Haque et.al. (2014) suggested the interaction between emotions and behavior may be manifested in several aspects within the TRA, as they may act as both antecedents and mediators. Hence, emotions may act as mediators in the TRA, as the cognitive assessment of the situation (attitudes) may evoke emotions, which in turn may affect behavioral choice [17]. Emotions are also found to be linked with social norms. Harland et al. (1999) described personal norms, stating that “personal norms reflect commitment with internalized values and are experienced as feelings of personal obligation to engage in a certain behavior” [18]. According to Elster (1994), when people obey norms, they often expect to avoid others’ disapproval. On the one hand, emotions are involved in all social norms as internal or external enforcers, but on the other, social norms may regulate the expression of emotions and sometimes the emotions themselves. Emotional reactions to emotional states are, therefore, often mediated by social norms [19].

During times when a radical behavior change is required, one that is accompanied by negative emotions such as loneliness and/or fear, social support may act as a soothing factor. Social support is defined as the exchange of resources among at least two people, perceived by both giver and recipient as a resource designed to increase the recipient’s quality of life [20]. This can be tangible or abstract, helping an individual and protecting them from the adverse effects of stressful life events [21], and was found to mediate loneliness and depression among older people [22].

Among Israeli COVID-19 patients in 2020, about 75% of the seriously ill were over 60, while their proportion in the population was only 15.8% [23].

To prevent the public from contracting the virus, the Israeli Ministry of Health (MOH) followed the WHO’s guidelines, focusing on instructions aimed at minimizing contact and maintaining optimal hygiene, mainly physical distancing, otherwise referred to as social distancing. This was directed at the overall population, but considered extremely important for people over 60 [24].

The current study aimed at assessing older Israelis’ distancing adherence and negative emotional levels, as well as identifying the factors associated with them. The study assumed that while adhering to distancing instructions among the elderly population was crucial in preventing morbidity and mortality, it may also be associated with an elevated risk of emotional harm due to loneliness.

The study’s hypotheses are:

- Adherence to distancing instructions will be associated with attitudes, subjective norms, and threat perception regarding COVID-19. Higher threat perception and more positive attitudes and subjective norms towards adherence to instructions will be associated with better adherence.
- Emotional levels will be associated with levels of social support. Higher social support may regulate negative emotions.
- Attitudes and subjective norms will act as mediating factors in the association between threat perception, trust and social support, and distancing adherence and negative emotions.

## 2. Materials and Methods

This study was a cross-sectional online survey. Data collection was conducted using a snowball sample via links provided on social networks. Data were collected between 19–21 April 2020—a period of home quarantine due to the COVID-19 epidemic in Israel (The first home quarantine due to the COVID-19 epidemic in Israel took place between 15 March and 4 May). Participants were not compensated for participating in the study.

The study was conducted according to the Declaration of Helsinki guidelines, and approved by the Institutional Ethics Committee of the Max Stern Yezreel Valley College (approval ref. EMEK YVC 2020-68).

The participants were 1822 Israeli citizens above the age of 60 living in the community. They were 60 to 96 years old (M = 69, *SD* = 6.01). Two-thirds were female (66.4%), 79.5% were Israeli-born, and most were Jewish (87.3%). Most respondents were married or had a meaningful relationship (76.5%), and almost all had children (96.8%). Two-thirds (63.3%) lived in urban environments, 57% were secular, and 55.5% had an academic education. Most were retired (60%) and reported a good to excellent economic status (60.6%).

### 2.1. Measures

The study questionnaire included 76 items and was distributed in both Hebrew and Arabic. It was based upon existing valid questionnaires, adjusted to this study’s subjects and target population [9,25,26,27,28,29].

The dependent variables were *adherence to distancing instructions* and *negative emotional level*. Negative emotions are defined by the American Psychological Association (APA) as “an unpleasant, often disruptive, emotional reaction designed to express a negative affect”.

To measure the adherence, we used four items as detailed in Table 1 (Cronbach’s alpha = 0.69). Negative emotional level was measured using the Positive and Negative Affect Schedule (PANAS) [28]. We computed this variable based on seven negative emotions as detailed in Table 1 (Cronbach’s alpha = 0.91).

Five primary independent variables were used as described below, and background characteristics were collected.

*Trust in the health care system* was defined as a “set of expectations that patients have from the healthcare system to help them heal” [30]. In the current study, this was measured based on the Multidimensional Trust in Health-Care Systems Scale (MTHCSS) questionnaire [25]. We used the following three items (Cronbach’s alpha = 0.70) to measure the respondents’ trust: “I have full confidence in physicians”; “The Israeli healthcare system does a good job”; and “I trust the medical information published by the MOH completely”. Response options ranged from “strongly disagree” (1) to “strongly agree” (7). The use of the above three items from the MTHCSS had been validated previously [31].

*Attitudes and subjective norms towards distancing* were measured using Fishbein and Ajzen’s questionnaire adjusted for the current study [9]. The respondents’ *attitudes* were measured using four items (Cronbach’s alpha = 0.80): “I believe that keeping a physical (social) distance is important to my health”; “I believe that keeping a physical (social) distance protects me from morbidity and implications of the new Corona disease”; “In my opinion, it’s important to follow the instructions of the MOH regarding physical (social) distancing”; and “I don’t believe in the importance of keeping a physical (social) distance”. Response options ranged from “strongly disagree” (1) to “strongly agree” (7).

*Subjective norms* were measured using four items (Cronbach’s alpha = 0.87): “Most of my family members believe in the importance of keeping a physical (social) distance”; “Most of my friends believe in the importance of keeping a physical (social) distance”; “Most of my friends follow the instructions for keeping a physical (social) distance”; and “Most of the people that are important to me and whose opinion I value believe in the importance of keeping a physical (social) distance”. Response options ranged from “strongly disagree” (1) to “strongly agree” (7).

*Social support* was measured using the Multidimensional Scale of Perceived Social Support (MSPSS) questionnaire [29]. The questionnaire included items such as: “There is a special person who is around when I am in need” and “I have friends with whom I can share my joys and sorrows”. Response options ranged from “strongly disagree” (1) to “strongly agree” (7). Internal consistency for the 11 items in our sample was high (Cronbach’s alpha = 0.85).

*The threat perception* measure was based on the HBM subscale of the perceived threat. Three items were used (Cronbach’s alpha = 0.70): “The thought that I’ll be sick with the Corona disease frightened me”; “The Corona virus might cause complications and even death”; and “If I’m sick with the Corona disease it would be hard for me to function”. Response options ranged from “strongly disagree” (1) to “strongly agree” (5).

### 2.2. Data Analysis

Data were analyzed using SPSS version 26. Background variables were described by frequencies and percentages, means, and standard deviations. Means, standard deviations, and inter-correlations for the study variables were calculated. The associations between the dependent variables and the background variables were analyzed with a series of t-tests, analyses of variance, and Pearson correlations.

The study model was examined with a path analysis using AMOS (version 26). Background variables served as control variables and were either continuous or binary. All continuous variables were standardized. The control variables, the independent variables, the mediators, and the dependent variables were allowed to correlate within themselves. χ^2^, *NFI*, *NNFI*, *CFI*, and *RMSEA* were used as model fit measures. The model was calculated using bootstrapping with 5000 samples, and bias-corrected confidence intervals with 95% confidence level. In order to interpret the significant mediating effects, the Monte Carlo method [32] was used, which requires a significant effect from the independent measure to the mediator and from the mediator to the dependent measure. This was used to construct confidence intervals for each indirect effect by reporting lower-level and upper-level values. Due to the large sample size, the significance level was set at *p* < 0.01.

## 3. Results

Most respondents reported good to excellent health. *Distancing behavior* was rated as high, yet only about 67% reported that none of their family members visited them at home during the period of the most severe lockdown instructions (Table 1). Adhering to distancing instructions was unrelated to age or gender, and was slightly higher among married than among single respondents (*p* = 0.003). It was higher among secular and partly religious participants than among religious participants (*p* < 0.001). It was higher among participants living in the city or in community settlements than among those living in rural settlements (*p* < 0.001), and it was higher among participants with a professional or an academic education than among participants with a high school education (*p* < 0.001).

Negative emotions were slightly associated with age (*p* = 0.005), and higher among females than among males (*p* < 0.001). They were higher among participants with a high school education than among participants with a professional or academic education (*p* < 0.001), and higher among participants who were unemployed than among those who were either employed or retired (*p* < 0.001). They were higher among participants with a low to reasonable economic status than among participants with a good to excellent economic status (*p* < 0.001).

Adherence to distancing instructions was positively associated with the perception of threat, attitudes, and subjective norms. Negative emotions were positively associated with the perception of threat, and negatively associated with social support, attitudes, and subjective norms (Table 2).

**Figure 1:**
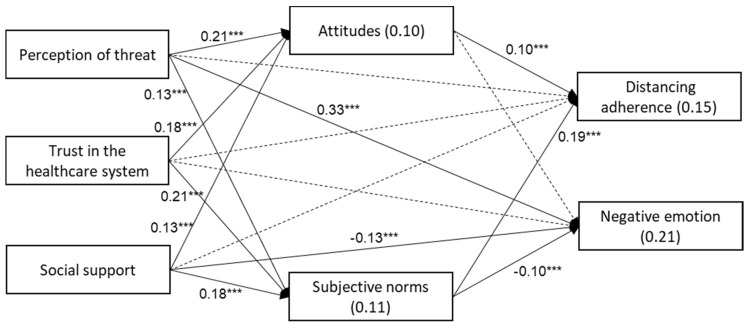
Path analysis for the perception of threat, trust in the healthcare system, social support, attitudes, and subjective norms towards following distancing instructions and the behavior of following social distancing and negative feelings. *** *p* < 0.001. Note: the control variables were excluded from the figure for purposes of clarity. Values on arrows—β values; values within the rectangles—*R*^2^.

The study model was examined with a path analysis (Figure 1). The control variables were: age, gender (1–male, 0–female), marital status (1–married or in a relationship, 0–other), place of living (1–urban, 0–rural), economic status (1–good or excellent, 0–low to reasonable), and health status (1–very good or excellent, 0–low to good). The independent variables were the perception of threat, trust in the healthcare system, and social support. The mediating variables were attitudes and subjective norms towards distancing, and the dependent variables were distancing adherence and negative emotions. The model was found to fit the data: χ^2^(28) = 29.18, *p* = 0.403, *NFI* = 0.988, *NNFI* = 0.999, *CFI* = 0.999, *RMSEA* = 0.005.

As shown in Table 3 and Figure 1, distancing adherence was positively associated with attitudes and subjective norms. Negative emotions were positively associated with the perception of threat and negatively associated with social support and subjective norms. Both attitudes and subjective norms were positively associated with the perception of threat, trust in the healthcare system, and social support. Analysis of the general indirect relationships concerning both attitudes and subjective norms revealed several significant relationships (analyzed within the path analysis) at *p* < 0.001, as shown in Table 4.

Using the Monte Carlo method to interpret the specific mediated relationships, the attitudes were found to mediate the relationships between the perception of threat and distancing behavior (95% CI = 0.009, 0.034), between trust and distancing behavior (95% CI = 0.008, 0.029), and between social support and distancing behavior (95% CI = 0.006, 0.023). Likewise, subjective norms were found to mediate the relationships between the perception of threat and distancing behavior (95% CI = 0.014, 0.034), between trust and distancing behavior (95% CI = 0.026, 0.055), and between social support and distancing behavior (95% CI = 0.002, 0.048).

Subjective norms regarding distancing instructions were found to mediate the relationships between threat and negative emotions among the study population (95% CI = −0.022, −0.006), between trust and negative emotions (95% CI = −0.034, −0.010), and between social support and negative emotions (95% CI = −0.029, −0.009). Attitudes toward distancing were unrelated to negative emotions, and thus did not mediate its relationships with the independent variables. In sum, higher perception of threat, higher trust in the healthcare system, and higher social support were associated with more positive attitudes and subjective norms towards distancing instructions, which were in turn associated with greater distancing adherence. Further, higher perception of threat, higher trust in the healthcare system, and higher social support were associated with more positive subjective norms, which were in turn associated with a lower level of negative emotions.

## 4. Discussion

Since older people are considered at high risk for COVID-19 morbidity and mortality, adhering to distancing instructions is of the utmost importance. However, while adhering to these instructions is crucial, it may also be associated with an elevated risk of emotional harm due to fear, stress, and loneliness. The current study was aimed at assessing distancing adherence and negative emotional levels of older Israelis, and identifying the factors associated with them.

### 4.1. Adhering to Distancing Instructions

Overall, we found distancing adherence to be high. High adherence was also demonstrated in other countries and among varied age groups [4,33]. However, while most of the respondents in the current study reported adhering to distancing instructions, more than a third reported allowing family members’ visitations.

Unlike previous studies [10,34], we did not find age, gender, or health status to predict distancing adherence. This may be attributed to the fact that this study was conducted during a uniquely severe health crisis, threatening all members of this at-risk population.

The current study hypotheses were that adherence to distancing instructions would be associated with attitudes, subjective norms, and perceived threat regarding COVID-19. Furthermore, we hypothesized that attitudes and subjective norms would act as mediating factors in the association between threat, trust, and social support, and distancing adherence.

In concurrence with the TRA [8], this study found attitudes and perceived social norms to be fundamental in predicting distancing adherence. The more positive the attitudes towards distancing and subjective norms were, the higher rates of distancing adherence reported. This was not surprising, and was strongly supported by previous studies examining attitudes and norms as predictors of adherence to various health behaviors [35,36,37,38].

The results of the present study further reveal that the perception of threat as defined by the HBM [26,39] assisted in shaping the respondents’ attitudes regarding distancing behavior. As expected, a higher perception of threat was associated with more positive attitudes towards distancing, which in turn were associated with better distancing adherence. Anxiety regarding the possibility of getting ill, and worry regarding the disease’s consequences, were linked to a more positive point of view regarding the effectiveness of distancing and better adherence to it. This was also supported by the Common-Sense Model of Self-Regulation, which explicates the processes by which people become aware of a health threat, navigate their affective responses to it, formulate perceptions of it and potential coping actions, create action plans for addressing it, and integrate continuous feedback on action plan efficacy and threat progression [40].

Finally, trust in the healthcare system was found to be instrumental. The higher the level of trust, the more positive the respondent’s attitudes were. In turn, more positive attitudes were associated with higher levels of perceived adherence in a person’s surroundings. In other words, it seemed that higher levels of trust were inevitably associated with better adherence.

It was not surprising that trust in government officials (in this case, the MOH) helped in shaping attitudes regarding the necessity of the instructions issued. The role of trust in the healthcare system, where adherence is concerned, had been previously examined. A review of the literature points to it as a critical predictor of medication adherence [41] and better health results [42].

### 4.2. Negative-Emotion Regulation

While it is well established that perceptions, attitudes, and norms may shape behavior, only a few studies have discussed the association between these variables and a person’s emotional state. The current study hypothesized that attitudes and subjective norms would act as mediating factors in the association between threat perception, trust, and social support, and negative emotions, and that the emotional levels would be associated with the respondents’ reported levels of social support.

These results highlighted the importance of subjective norms in the regulation of negative emotions among older people during the COVID-19 pandemic. As described in the Results section, higher levels of perceived norms; i.e., a sense of prevalent adherence among people nearby, or in other words, a perception of “we are all in the same boat”, assisted in regulating negative emotions. This may be attributed to people’s social need to feel similar to others around them. Humans have a basic need to belong, and to form and maintain interpersonal relationships [43]. Subjective norms and the behavior of people around them may promote a sense of being part of a close-knit group, thus helping to control levels of emotional stress, as the feeling that “most of my friends are social distancing” may reduce tension, stress, and fear. As mentioned by Elster (1994), social norms may regulate the expression of emotions, and sometimes even the emotions themselves [19].

Furthermore, the results of the current study showed that, as expected, a higher perception of threat was associated with higher levels of negative emotions. This points to the need for careful balancing regarding the use of intimidation strategies as a public health tool designed to promote adherence. While intimidation strategies have been known to promote adherence, they also have the potential of evoking negative emotions, thus harming the population’s wellbeing [44,45,46,47].

The current study may offer a way of regulating levels of negative emotions, emphasizing the importance of social support during these times. Social support has previously been shown to be directly related to improved wellbeing, as well as serving as a buffer against potentially adverse effects [48]. Our findings further supported this, suggesting that higher levels of social support were associated with lower reported negative emotions. Therefore, maintaining social support during social distancing is of the utmost importance for older people’s wellbeing. As mentioned previously, the importance of social support in general, and specifically among older people, is repeatedly mentioned in the literature. Previous studies showed that adults and elderly people relied mostly on spouses, followed by family and then friends for social support [49].

As most countries are in the midst of coping with ongoing morbidity caused by new COVID-19 strains, and vaccinations, while progressing, are yet unable to provide a complete solution, adhering to social distancing remains crucial in maintaining the health of the older population. To this end, understanding social-distancing adherence among the elderly and their wellbeing during these times is of the utmost importance.

Countries around the world faced common challenges while coping with the COVID-19 pandemic. However, the pandemic found Israel in the midst of recurrent election campaigns that may have influenced the way it was managed. Dealing with the pandemic enabled politicians to present themselves as problem solvers and leaders. In order to enhance the public’s trust, our recommendation is to use health professionals to create and convey uniform, reliable messages, as well as to keep the conversation regarding the pandemic clean of political interests. Health messages should be tailored to specific populations according to their characteristics. This culturally adjusted approach may also promote the population’s trust.

The current study may be subject to several limitations: data were self-reported and have a probability of recall bias [50]. As the questionnaire was distributed via smartphones, our sample was limited to people with smartphones and the ability to use them (functioning internet and adequate technological literacy). In addition, data were collected through snowball sampling, rather than a representative sample, and due to the cross-sectional design, we were unable to learn about the process of forming and regulating negative emotions through time. Furthermore, while we assumed that lockdown had probably led to loneliness, we did not measure it directly, and instead focused on negative emotions.

However, the large sample size was rapidly collected, amidst the most intense period of quarantine, which allowed us to cautiously assess distancing adherence and learn about the population’s wellbeing.

## 5. Conclusions

While the importance of the clinical aspects is indisputable when coping with a pandemic, its effect on the population’s wellbeing should not be overlooked. Efforts should be invested in addressing the reliability of messages regarding COVID-19 and discovering creative ways of raising social support during lockdowns.

Our conclusions highlight several applicable aspects that should be considered:

It is imperative to find ways to maintain and promote higher levels of social support among the older population during distancing periods. This may be done through official channels, such as government programs (for example, by allocating funds for more social workers); as well as by encouraging local initiatives, such as volunteers who keep in touch with older people who are known to be living alone.

The fundamental part that trust plays in both distancing adherence and regulating negative emotions points to the importance of addressing this issue when interventions aimed at improving adherence are designed.

## Figures and Tables

**Table 1:** Adherence with the instructions for physical/social distancing and negative emotions among the study population (*N* = 1822).

	Mean (SD), Range 1–5	% of Respondents Who Responded 4 or More
**Adherence**	4.30(0.81)	___
Family members visited me at home	1.76(1.22)	0.15
Neighbors or friends visited me at home	1.34(0.88)	0.059
I avoided meeting family members	4.09(1.20)	0.742
I avoided meeting neighbors or friends	4.22(1.15)	0.789
	**Mean (SD), Range 1–7**	**% of Respondents Who Responded 5 or More**
**Negative Emotion**	2.86(1.41)	___
Frustrated	3.20(1.87)	0.283
In a bad mood	3.05(1.73)	0.252
Stressed	3.02(1.78)	0.248
Angry	2.81(1.74)	0.211
Anxious	2.74(1.76)	0.205
Nervous	2.73(1.70)	0.186
Frightened	2.46(1.66)	0.156

**Table 2:** Means, standard deviations, and inter-correlations for the study variables (*N* = 1822).

	*M* (*SD*)	2.	3.	4.	5.	6.	7.
1. Adherance (1–5)	4.30 (0.81)	−0.01	0.12 ***	0.04	0.02	0.22 ***	0.26 ***
2. Negative emotions (1–7)	2.86 (1.41)		0.34 ***	−0.03	−0.16 ***	−0.08 **	−0.13 ***
3. Perception of threat (1–5)	3.61 (0.99)			0.02	0.02	0.21 ***	0.13 ***
4. Trust in the healthcare system (1–7)	4.82 (0.81)				0.08 **	0.20 ***	0.22 ***
5. Social support (1–7)	5.96 (0.96)					0.15 ***	0.21 ***
6. Attitudes (1–7)	5.95 (1.12)						0.58 ***
7. Subjective norms (1–7)	6.06 (0.89)						

** *p* < 0.01, *** *p* < 0.001.

**Table 3:** Direct relationships among threat, trust, social support, attitudes, and subjective norms towards distancing adherence and negative emotions (*N* = 1822).

*DV* (*R*^2^)	*IV*	β	SE	*p*
Attitudes towards distancing instructions (0.10)	Perception of threat	0.21	0.02	<0.001
	Trust in the healthcare system	0.18	0.02	<0.001
	Social support	0.13	0.02	<0.001
Subjective norms regarding distancing instructions (0.11)	Perception of threat	0.13	0.02	<0.001
	Trust in the healthcare system	0.21	0.02	<0.001
	Social support	0.18	0.02	<0.001
Distancing adherence (0.15)	Perception of threat	0.05	0.02	0.018
	Trust in the healthcare system	−0.03	0.02	0.188
	Social support	−0.05	0.02	0.038
	Attitudes	0.10	0.03	<0.001
	Subjective norms	0.19	0.03	<0.001
Negative emotions (0.21)	Perception of threat	0.33	0.02	<0.001
	Trust in the healthcare system	0.03	0.02	0.211
	Social support	−0.13	0.02	<0.001
	Attitudes	−0.06	0.03	0.032
	Subjective norms	−0.10	0.03	<0.001

Note: *DV* = dependent variable; *IV* = independent variable.

**Table 4:** Indirect relationships among threat, trust, and social support, and distancing adherence and negative emotions (*N* = 1822).

*DV* (*R*^2^)	*IV*	Standardized Indirect Effect	*SE*	95% CI
Distancing adherence (0.15)	Perception of threat	0.046	0.008	0.034, 0.063
	Trust in the healthcare system	0.059	0.009	0.042, 0.077
	Social support	0.047	0.007	0.031, 0.060
Negative emotions (0.21)	Perception of threat	−0.025	0.005	−0.036, −0.015
	Trust in the healthcare system	−0.032	0.006	−0.044, −0.021
	Social support	−0.026	0.005	−0.035, −0.016

Note: *DV* = dependent variable; *IV* = independent variable.

## Data Availability

Data supporting the reported results can be accessed through the corresponding author.
